# Interrelations between Iron and Vitamin A—Studied Using Systems Approach

**DOI:** 10.3390/ijms23031189

**Published:** 2022-01-21

**Authors:** Kaja Gutowska, Dorota Formanowicz, Piotr Formanowicz

**Affiliations:** 1 Institute of Computing Science, Poznan University of Technology, 60-965 Poznan, Poland; Kaja.Gutowska@cs.put.poznan.pl; 2 Department of Medical Chemistry and Laboratory Medicine, Poznan University of Medical Sciences, 60-806 Poznan, Poland; doforman@ump.edu.pl; 3 Institute of Bioorganic Chemistry, Polish Academy of Sciences, 61-704 Poznan, Poland

**Keywords:** iron homeostasis, iron deficiency, vitamin a deficiency, modeling, petri nets, t-invariants

## Abstract

A deficiency of vitamin A (VAD) and iron is the most common nutritional problem affecting people worldwide. Given the scale of the problem, the interactions between vitamin A and iron levels are widely studied. However, the exact mechanism of the impact of vitamin A on the regulation of iron metabolism remains unclear. An extremely significant issue becomes a better understanding of the nature of the studied biological phenomenon, which is possible by using a systems approach through developing and analyzing a mathematical model based on a Petri net. To study the considered system, the t-cluster analysis, the significance analysis, and the analysis of the average number of transition firings were performed. The used analyses have allowed distinguishing the most important mechanisms (both subprocesses and elementary processes) positively and negatively regulating an expression of hepcidin and allowed to distinguish elementary processes with a higher frequency of occurrence compared to others. The analysis also allowed to resolve doubts about the discrepancy in literature reports, where VAD leads to positive regulation of hepcidin expression or to negative regulation of hepcidin expression. The more detailed analyses have shown that VAD more frequently positively stimulates hepcidin expression and this mechanism is more significant than the mechanism inhibiting hepcidin expression indirectly by VAD.

## 1. Introduction

### 1.1. Research Context

Considerations regarding the role of vitamin A deficiency (VAD) in the regulation of hepcidin expression and, consequently, VAD effect on iron metabolism were (and still are) undertaken by many researchers [1,2,3,4,5,6]. The first literature reports on the relationship between vitamin A deficiency and low iron levels come from studies conducted in the 1920s [7,8,9]. Additionally, vitamin A and iron deficiencies are the most common nutrient deficiencies affecting millions of people around the world [1,3,5,6]. However, the mechanism of vitamin A influence on iron homeostasis was not fully understood [2,6]. Moreover, the study of these relationships raises a lot of controversy among researchers, which can be observed by the discrepancies in the literature reports. With the purpose to understand the rules underlying this phenomenon, a systems approach based on Petri nets [10,11,12] was used and described in this paper. This approach allows the systematization of knowledge and a better understanding of the nature of the biological system through creating a mathematical model being a representation of the complex network of interactions [13].

### 1.2. Biological Background

Iron is a unique trace element that is biologically essential, but also potentially toxic for humans, hence, maintaining its homeostasis is an extremely important issue. The essence of intracellular iron homeostasis is providing adequate amounts of iron to maintain the continuity of vital biological processes, as well as limiting the toxicity of Fe${}^{2+}$ ions involved in the Fenton reaction [14].

Iron differs from other minerals because, due to the lack of a physiological excretion mechanism, its balance in the body is tightly controlled at the level of absorption. Iron ions in the form of Fe${}^{3+}$ are reduced to Fe${}^{2+}$ by duodenal cytochrome b (Dcytb), and in the next stage, they are transported to the enterocyte by the divalent metal transporter 1 (DMT1). Then, Fe${}^{2+}$ ions can be absorbed by ferritin (Ft), or they can be transported into the circulation via ferroportin (FPN) using hephaestin (Heph). Heph oxidizes Fe${}^{2+}$ ions to Fe${}^{3+}$, which are then absorbed by mammalian cells [15]. Newly absorbed Fe${}^{3+}$ binds to plasma transferrin (Tf). Iron-loaded Tf binds to transferrin receptor 1 (TfR1) on the surface of most of the cells and forms a complex. The Tf-TfR1 complex is internalized via clathrin-mediated endocytosis [15]. Next, after endocytosis of this complex, iron enters the cytoplasm via DMT1. If iron is needed it may traffic directly to the sites of utilization, like mitochondria. If there is no requirement for iron, it may be sequestered for later use within ferritin—the iron-storage protein. The cell can also get rid of iron by exporting via FPN. This export pathway can act as a safety valve if very large amounts of iron accumulate in the cell. In enterocytes, the efficiency of cellular iron export is enhanced by Heph, but in most cells of the body, this role is played by the circulating Heph homolog, ceruloplasmin [15]. An important element is the cytoplasmic labile iron pool (LIP), indicating a deficiency or excess of iron. LIP in response to abnormal iron levels, activates the pathways regulating iron homeostasis [16]. Iron metabolism is controlled by two regulatory mechanisms: the first one acts at the cellular level, while the other works at the systemic level.

The regulatory mechanisms at the cellular level are based on the iron regulatory protein (IRP)—iron responsive element (IRE) signaling pathway. They involve cytoplasmic regulatory proteins 1 and 2 (IRP1 and IRP2) that bind to specific RNA sequences, iron-reactive sequences (IREs), thus regulating the expression of proteins involved in the maintenance of homeostasis, like TfR1, DMT1, Ft and FPN [15]. Depending on the cellular iron levels the IRP/IRE mechanism controls the synthesis of these proteins and their action. For the detailed IRP/IRE signaling pathway translation modulation mechanisms see [17].

Iron deficiency induces the formation of apo-IRP1, which binds to the IRE sequence in the 5′UTR region of the Ft and Fpn mRNAs, leading to an inhibition of their translation. On the other hand, the binding of apo-IRP1 to the IRE sequence in the 3′UTR mRNA region of TfR1 and DMT1 increases the encoded proteins. Otherwise, excess iron will induce the formation of holo-IRP1, which has an opposite effect. It inhibits the translation of TfR1 and DMT1 and increases the level of Fpn and Ft proteins [18].

The regulatory mechanism functioning at the iron system homeostasis level depends mainly on hepcidin (a hormone produced in the liver), the master regulator of systemic iron homeostasis [19]. The major stimuli regulating hepcidin transcription include iron concentrations in the blood, liver iron stores, inflammation, and erythropoiesis [20]. Hepcidin binds to FPN (in the enterocyte membrane), and as a consequence, the Janus kinase 2 (Jak2) is attached. It leads to the phosphorylation of tyrosine (Tyr) residues in FPN, which signals the protein translocation from the membrane into the cytoplasm. As a consequence, FPN is dephosphorylated, ubiquitinated to be degraded eventually. Because of the FPN degradation, iron becomes blocked in enterocytes, decreasing the amount of iron transported to the blood [18].

Hepcidin production is stimulated by iron loading and inflammation. Iron-mediated hepcidin regulation occurs via the bone morphogenetic protein (BMP) and SMAD (BMP-SMAD) pathway, whereas inflammation-mediated regulation occurs in both the IL-6 and JAK and signal transducer and activator of transcription proteins (STAT) (IL-6/JAK/STAT) signaling axis and via the BMP-SMAD pathway [19].

One of the hepcidin regulation mechanisms involves Tf and hemochromatosis (HFE)—proteins, which compete for the binding of the membrane TfR1 [1]. In normal iron states or excess serum iron, Tf binds with a high affinity to TfR1, thus preventing the binding of HFE to TfR1. Free HFE sends signals to the hepatocyte nucleus increasing hepcidin expression. In turn, in the case of iron deficiency, HFE binds to TfR1, reducing the expression of hepcidin [19].

Hepcidin expression can be also modulated by the inflammatory processes [20,21]. Overproduction of cytokines such as IL1β, TNF-α and IL-6 by macrophages and IFN-γ by lymphocytes weakens erytropoietin (EPO) synthesis, reduces the response to erythropoiesis, increases hepcidin levels and may activate erythrophagocytosis, especially in an acute form [19]. IL-6 induces the expression of the Hamp gene through the JAK and the signal transducer and activator of the transcription 3 (STAT3) pathway (JAK-STAT3). IL-6 is bundled up with JAK protein triggering its autophosphorylation and activation, resulting in phosphorylation of the transcription factor STAT3. In turn, STAT3 is translocated to the cell nucleus, where leads to the stimulation of the promoter of the hepcidin gene, increasing the expression of hepcidin [19].

The hemojuvelin—bone morphogenetic protein 6—and signaling protein 4 (HJV-BMP6-SMAD4) pathway is another way to modulate hepcidin expression [22]. HJV is an essential cofactor for the binding of bone morphogenetic protein 6 (BMP6) to the BMP receptor (BMPRs), leading to the phosphorylation of the signaling proteins SMAD 1, 5, and 8 (nuclear signal transducers). Phosphorylated SMAD 1, 5, and 8 proteins form a complex to bind the SMAD4 protein in the next step, which is a signal to move to the nucleus and stimulate transcription of the hepcidin gene [23]. This mechanism is triggered in the event of an excess of iron.

In iron deficiency, the HJV-BMP6-SMAD4 pathway is inhibited by furin, specifically, by the action of the soluble form of hemojuvelin (sHJV). sHJV is released from the cell membrane by furin and then binds to the BMP6 protein, preventing it from binding to the receptor. Consequently, the HJV-BMP6-SMAD4 pathway is inhibited, and thus, the concentration of hepcidin is reduced [16].

Besides, hepcidin expression is modulated by VAD. It turns out that vitamin A may increase or decrease hepcidin expression. VAD is characterized by an increase in the level of the pro-inflammatory cytokine, such as IL-6 and IL-1β, responsible for increasing hepcidin expression [1]. VAD also increases the level of the BMP6 protein, which is essential in the HJV-BMP6-SMAD4 pathway that stimulates the transcription of the hepcidin gene [2]. On the other hand, the level of the BMP6 protein is reduced in the iron deficiency state [2], reducing the hepcidin expression by preventing the HJV-BMP6-SMAD4 pathway. An equally important mechanism for modulating hepcidin expression is the impairment of erythropoiesis, both in the case of iron deficiency and vitamin A deficiency [2]. VAD leads to impaired erythropoiesis by lowering erythropoietin expression. It leads to erythrocyte malformations and, consequently, accumulation of the heme group in the spleen [2].

**Figure 1:**
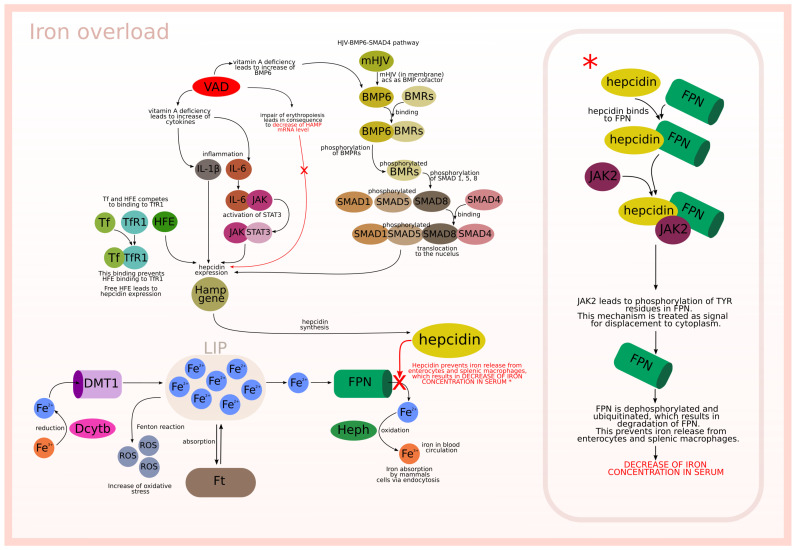
Iron overload.

**Figure 2:**
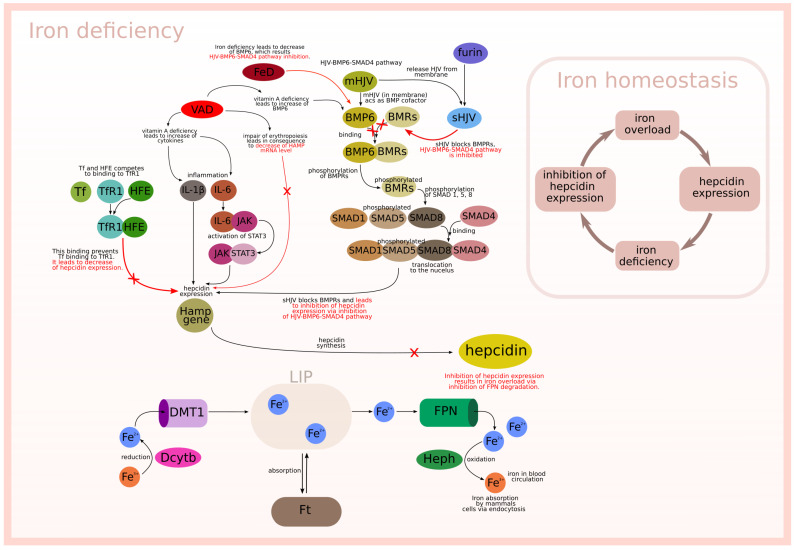
Iron deficiency.

The described biological background is graphically presented in Figure 1 and Figure 2, which relate to two different situations. Figure 1 is focused on the interactions between modeled subprocesses in an iron overload situation, while Figure 2 concerns the interactions between the modeled subprocesses in an iron deficiency situation.

## 2. Results

### 2.1. Petri Net-Based Model

**Figure 3:**
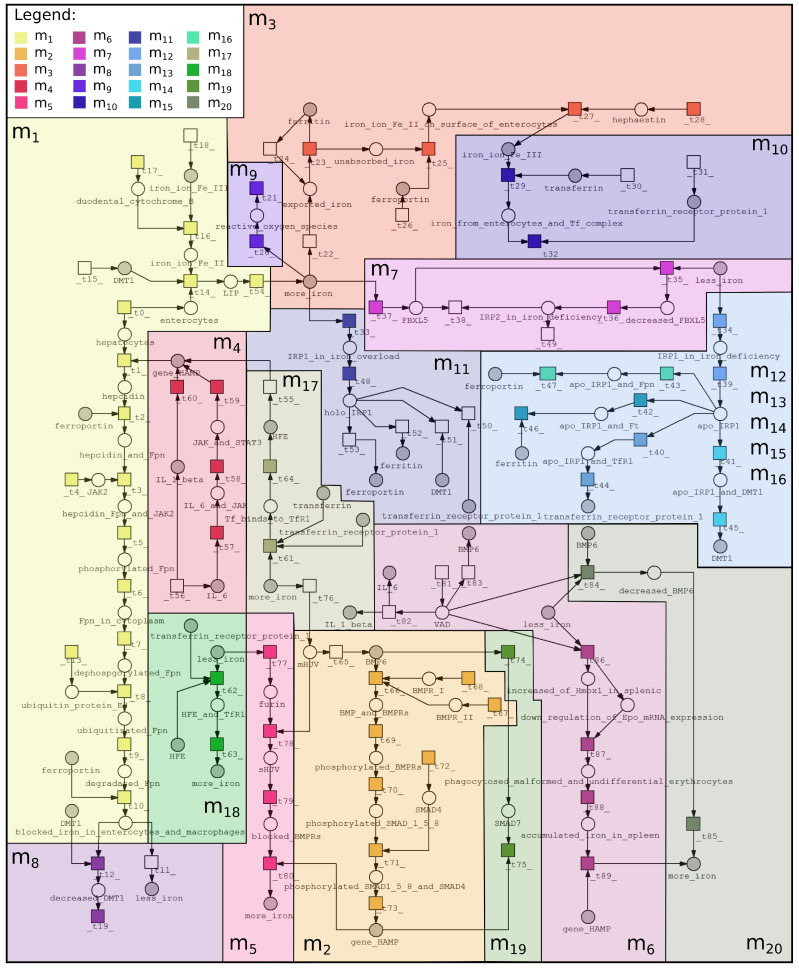
Petri net-based model of interrelations between vitamin A and iron was divided into 20 subprocesses according to MCT sets: $m_{1}$—iron export to enterocyte, $m_{2}$—expression of hepcidin by HJV-BMP6-SMAD4 pathway, $m_{3}$—iron absorbtion by Ft, $m_{4}$—expression of hepcidin by cytokine IL-6 and IL-1β, $m_{5}$—inhibition of hepcidin expression via soluble HJV, $m_{6}$—impairment of erythropoiesis, $m_{7}$—decrease of Ft and Fpn (iron deficiency), $m_{8}$—degradation and internalization of Fpn, $m_{9}$—fenton reaction, $m_{10}$—iron absorption by mammalian cells, $m_{11}$—holo-IRP1 formation (increase of iron concentration), $m_{12}$, $m_{13}$, $m_{14}$, $m_{15}$ and $m_{16}$—Apo-IRP1 formation (decrease of iron concentration), $m_{17}$—increase of hepcidin expression (normal iron status and iron overload), $m_{18}$—decrease of hepcidin expression (iron deficiency), $m_{19}$—negative regulation of hepcidin by SMAD7, $m_{20}$—inhibition of HJV-BMP6-SMAD4 pathway (iron and vitamin A deficiency). More detailed descriptions of these subprocesses are included in Table 2, where information about transitions contained in each MCT set is also included.

The purpose of creating a model based on Petri nets is to systematize knowledge and better understand the interrelations between vitamin A deficiency and iron deficiency. Although vitamin A deficiency affects the regulation of iron homeostasis, these mechanisms remain unclear. This fact became a motivation to focus on this biological phenomenon. The proposed Petri net-based model was created using Snoopy (open-source software) [24], while the analysis was performed using Holmes (free, stand-alone Java application) [25]. Properties of the proposed model are as follows: 68 passive components (places), 89 active components (transitions), 402 t-invariants (subprocesses). The Petri net-based model was illustrated in Figure 3, which includes only names of places, while names of transitions are included in Table 1 (to increase the readability of the model). The model is available in Supplementary Materials.

### 2.2. Analysis

The analysis of the Petri net model is based on a search for similarities between t-invariants (subprocesses), leading to an identification of subprocesses that may interact with each other. Such knowledge allows for a better understanding of the modeled system, and may also allow for a discovery of interesting properties of the investigated biological system. The analysis of the proposed model includes a Maximal Common Transition (MCT) sets analysis, a t-cluster analysis, a significance analysis with the completion by a knockout analysis, and an analysis of an average number of firings of transitions (simulation). These methods are described in Section 4.

In the first stage of the analysis, the MCT sets analysis was performed. The proposed model contains 89 transitions (elementary processes), which were grouped into non-trivial MCT sets (see Section 4). In a biological context, MCT sets correspond to certain functional modules. Therefore, a biological meaning for each MCT set was assigned, what is described in Table 2. All the described modules are also marked in Figure 3 as colored frames.

**Figure 4:**
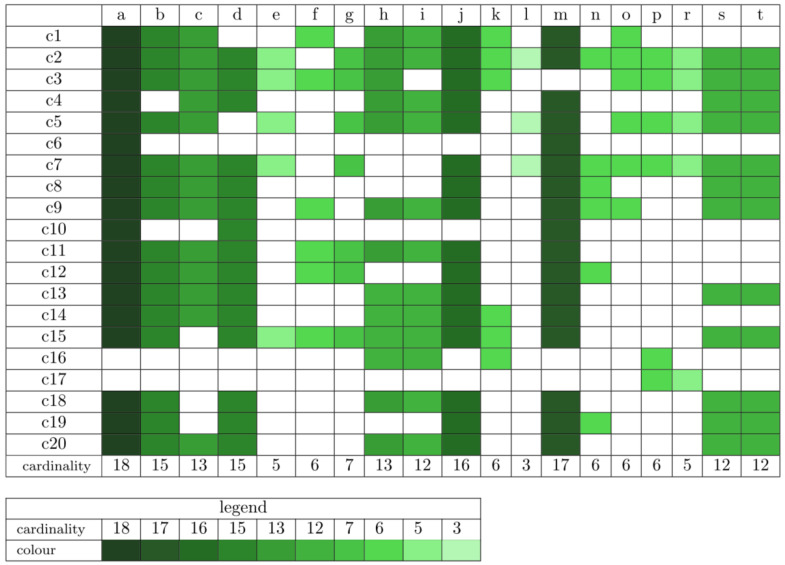
Occurrence of subprocesses in t-clusters (more intense the color, given subprocess appears in a greater number of t-clusters).

In the next stage, clustering of 402 t-invariants was performed. To assess the best clustering Mean Split Silhouette index (MSS) and Calinski–Harabasz (C–H) coefficient were used [22]. The first coefficient, MSS, evaluates the fit of each t-invariant to its cluster and the average quality of a given clustering [26,27]. The second one, C–H, allows indicating the optimal number of clusters by the highest value [28]. The set of t-invariants was divided into 20 t-clusters using Average Linkage method and Pearson distance measure. Each of these clusters consists of certain biological subprocesses, which are described in Table 3. Moreover, occurrences of the particular subprocesses in a given t-cluster are presented in Figure 4 (the more intense the color, the given subprocess appears in the greater number of t-clusters). Subprocesses, which may play a significant role in the modeled system, can be found through their frequency of occurrence in t-clusters.

The purpose of the t-cluster analysis is to find subprocesses that may play a significant role in the functioning of the modeled system. It is possible to identify subprocesses that may be more crucial than others. It seems that if a certain subprocess occurs in a larger number of clusters, it may be stimulated by various, independent processes. Due to this fact, it may be concluded whether this subprocess is stimulated more or less frequently. However, the analysis of t-clusters is quite general, and such conclusions need to be confirmed by applying more precise analyzes. For example, an essential mechanism of the investigated system is the regulation of hepcidin expression. The presented model includes several independent pathways involved in hepcidin expression, including HJV-BMP6-SMAD4 pathway (subprocess (n)), free HFE (subprocess (f)), and JAK-STAT3 pathway modulated by cytokines IL-6 and IL-1β (subprocess (h)). Therefore, the frequency of these subprocesses in all t-clusters was considered. As shown in Figure 4, subprocess (n) occurs in 6 t-clusters, subprocess (f) occurs in 6 t-clusters, and subprocess (h) occurs in 13 t-clusters. Therefore, it seems that hepcidin expression is more frequently induced by cytokines (subprocess (h)) than other pathways. However, this type of conclusion requires additional confirmation by conducting more detailed analyzes. The results of the t-cluster analysis are described in more detail in Section 3.

As mentioned before, the analysis of t-clusters may turn out to be quite general and thus may cause some phenomena to be ignored and not noticed. Therefore, it is also worth using more detailed analyzes, such as a significance analysis, a knockout analysis, or an analysis of the average number of transition firings (simulation). Conducting additional analyzes will allow to confirm the results obtained from the cluster analysis.

The significance analysis was performed for elementary processes related to hepcidin expression, both stimulation and inhibition. The results of the significance analysis for selected transitions are presented in Table 4. This table contains subprocesses that stimulate hepcidin expression and subprocesses that inhibit hepcidin expression (all subprocesses are listed in the “Subprocesses” column). Each subprocess consists of at least one elementary process; the transitions’ IDs and the names of elementary processes are included in the columns “ID” and “Name of elementary process”, respectively. The column, “Significance” is divided into “Frequency trans./t-inv.”, which includes information about the occurrence frequency of a given transition in supports of t-invariants, and “Percentage ratio” which includes occurrence frequency expressed as percentage, hereinafter called also significance of given transition. In addition, Table 4 includes also the results for the analysis of an average number of the firing of transitions, which are included in the last column “AvgT”. This analysis allows obtaining information about the frequency of transition activation. For example, transition $t_{59}$ (corresponding to hepcidin expression via the JAK-STAT3 pathway) is part of the larger subprocess of stimulating hepcidin expression by the cytokines IL-6 and IL-1β. This transition occurs in 252 supports of t-invariants which consists 63% of all t-invariants (402 t-invariants). The significance analysis is related to knockout analysis, which means that a knockout of transition $t_{59}$ excludes 63% of all modeled subprocesses. Additionally, the average number of firings of transition $t_{59}$ is equal to 46.94. This value is one of two the highest values among all elementary processes positively regulating hepcidin expression.

The significance analysis allows determining which elementary process is more significant for the functioning of the whole model. For example, based on Table 4, among the elementary processes that stimulate hepcidin expression, transitions $t_{59}$ and $t_{60}$ are the most important (these transitions correspond to elementary processes which positively regulate hepcidin expression by cytokines). In this case, these results confirm the conclusions of the t-cluster analysis, which has shown that hepcidin expression induced by cytokines (subprocess (h)) may be more significant than induction by other subprocesses. Moreover, another more detailed analysis is the analysis of the average number of firings of transitions. Contrary to the significance analysis, this is not a structure-based but a simulation-based analysis. Such simulation analysis allows obtaining information about the frequency of firings of a particular transition. For example, based on Table 4, transition $t_{60}$ is the most frequently fired among all elementary processes positively regulating hepcidin expression. Therefore, the elementary subprocess which is a positive regulation of hepcidin expression by cytokines occurs more frequently than other elementary processes positively regulating hepcidin. A detailed comparison of the results obtained from the mentioned analyses for the mechanisms stimulating and inhibiting hepcidin expression is described in Section 3.

## 3. Discussion

The proposed model is focused on the selected aspects of the interaction between vitamin A and iron, where different levels of iron concentration was taken into account (iron deficiency, normal iron status, iron overload). The performed analyses made it possible to study the interactions between particular subprocesses, and in consequence, a better understanding of the modeled phenomenon.

The analysis of t-clusters focused on the role of hepcidin in the regulation of iron homeostasis. There are several independent mechanisms and biological components influencing positively the expression of hepcidin, which are as follows:

1. HJV-BMP6-SMAD4 pathway (subprocess (n)) included in 6 t-clusters, see Table Figure 4).
2. Cytokines IL-6 (JAK-STAT3 pathway) and IL-1β (subprocess (h)) included in 13 t-clusters, see Table Figure 4).
3. Free HFE; in the case of iron overload or normal status Tf binds to TfR1 and prevents HFE binding (subprocess (f)) included in 6 t-clusters, see Table Figure 4).

Moreover, the cluster analysis showed that hepcidin expression induced by IL-6 and IL-β may be more significant than induction by other subprocesses. This fact can be associated with vitamin A deficiency, which additionally stimulates inflammation through an increase of the mentioned cytokines. However, other subprocesses, like subprocess (n) corresponding to HJV-BMP6-SMAD4 pathway and subprocess (f) corresponding to the situation in which there exists free HFE, occur in the same number of clusters. Therefore, the role of these subprocesses should be equally important for the functioning of the system. These results were compared to the results obtained from the more detailed analysis, which is the significance analysis (see Table 4). The results of the significance analysis showed that the most significant elementary processes are transitions associated with induction of hepcidin expression by cytokines ($t_{59}$, $t_{60}$ with a significance of 63%). On the other hand, the elementary processes related to the stimulation of hepcidin expression by HJV-BMP6-SMAD4 pathway and by free HFE have a similar significance level ($t_{73}$—29%, $t_{64}$—26%, respectively). Thus, the results of the significance analysis confirm the results of the t-cluster analysis for subprocesses related to the positive stimulation of hepcidin expression. The t-cluster analysis confirm the above results, because it has shown that subprocess (h) corresponding to induction of hepcidin expression by cytokines may be the most significant compared to the others, and subprocesses n) and f) although less crucial than subprocess (h), may be equally important for the functioning of the system. In addition to estimating the significance of selected transitions, an average number of firings of transitions was also considered (see the column “AvgT ” in Table 4). As can be seen, the most often fired are transitions $t_{59}$, $t_{60}$ and $t_{82}$ corresponding to stimulation of hepcidin expression through cytokines, next in order is transition $t_{73}$ corresponding to stimulation by HJV- BMP6-SMAD4 pathway, and finally transitions $t_{55}$ and $t_{64}$ corresponding to the presence of free HFE. Despite the fact that the last two elementary processes are equally important in the sense of the structure of the entire model, they may occur with different frequencies.

The next stage of the analysis of the results is focused on the mechanisms leading to inhibition of hepcidin expression, which are as follows:

1. Inhibition of HJV-BMP6-SMAD4 pathway (subprocess (o) included in 6 t-clusters and subprocess (s) included in 12 t-clusters, see Table 4).
2. Binding HFE to TfR1 (subprocess (g) included in 7 t-clusters, see Table 4).
3. Induction of SMAD7 by BMP6 (subprocess (k) included in 6 t-clusters, see Table 4).
4. Impairing of erythropoiesis (subprocess (t) included in 12 t-clusters, see Table 4).

The t-clusters analysis revealed that among the subprocesses inhibiting hepcidin expression, inhibition of HJV-BMP6-SMAD4 pathway and impairing of erythropoiesis are more relevant than the other mechanisms. HJV-BMP6-SMAD4 pathway can be inhibited by two different mechanisms. The first one is related to an activation of furin in case of iron deficiency. Furin releases HJV from a membrane as a result of a proteolytic reaction. Soluble sHJV blocks BMPRs receptors resulting in inhibition of HJV-BMP6-SMAD4 pathway. The second one is a reduction of BMP6 (in case of iron deficiency and VAD), which is an essential element in HJV-BMP6-SMAD4 pathway.

Similarly, as for the subprocesses related to the stimulation of hepcidin expression, a more detailed analysis was performed for the inhibitory mechanisms. The significance analysis has shown that the most significant elementary process is the transition associated with inhibition of HJV-BMP6-SMAD4 pathway ($t_{85}$ with 19% significance), next in order are elementary processes related to HFE binding to TfR1 ($t_{62}$ and $t_{63}$, both with 17% significance), followed by elementary processes related to SMAD7 induction by BMP6 ($t_{74}$ and $t_{75}$ with 10% significance), and finally elementary process corresponding to impairing of erythropoiesis ($t_{85}$ with 9% significance). These results of the significance analysis are not consistent with the t-cluster analysis. The t-cluster analysis has shown that subprocess (o) corresponding to inhibition of HJV-BMP6-SMAD4 pathway and subprocess (t) corresponding to impairing of erythropoiesis seem to be the most significant, while only the first one is really crucial. Moreover, subprocess (t) has the slightest importance in the significance analysis of the selected elementary processes. As mentioned earlier, the t-cluster analysis may be too general. It turns out that the most significant mechanisms inhibiting hepcidin expression are the inhibition of HJV-BMP6-SMAD4 pathway, as well as the binding of HFE and TfR1. Apart from the significance analysis, the simulation of the average number of firings was also performed. The fact that a given elementary process is significant from the point of view of the system structure does not mean that it is fired most often. In Table 4 in the column “AvgT ” it can be seen that induction of SMAD7 by BMP6 is most often fired (this mechanism indirectly inhibits HJV-BMP6-SMAD4 pathway, which requires the presence of BMP6). However, transitions $t_{74}$ and $t_{75}$ corresponding to the mentioned subprocess are not so much significant for the structure of the modeled system. The other mechanisms of inhibition of hepcidin expression (i.e., inhibition of HJV-BMP6-SMAD4 pathway or binding HFE to TfR1), which are structurally important, occur with much lower frequency than induction of SMAD7 by BMP6.

An important aspect of the model analysis is the effect of vitamin A deficiency and its relationship with iron. The cluster analysis identified a few subprocesses directly related to vitamin A deficiency based on the frequency occurrence of subprocesses in t-clusters:

1. VAD additionally stimulates hepcidin expression by increase of IL-6 and IL-1β (subprocess (i)) included in 12 t-clusters).
2. VAD leads to increase of BMP6 (subprocess (p) included in 6 t-clusters).
3. VAD and low iron level leads to decrease of BMP6, which results in inhibition of hepcidin expression (subprocess (s) included in 12 t-clusters).
4. VAD and iron deficiency impair of erythropoiesis leading to negative regulation of hepcidin expression (subprocess (t) included in 12 t-clusters).

The results of the t-cluster analysis (the frequency occurrence of the mentioned above subprocesses in t-clusters) revealed unquestionable influence of vitamin A deficiency and iron deficiency on inhibition of hepcidin expression. There exist different literature reports concerning the influence of vitamin A deficiency on hepcidin expression. The results presented in [1] clearly describe that vitamin A maintains iron homeostasis by positively modulating hepcidin expression in the liver. On the other hand, the results reported in [2] indicate that vitamin A deficiency also promotes reduction of hepatic Hamp mRNA levels, rather than the expected up-regulation of Hamp gene expression.

The experimental procedures of these two studies ([1,2]) leading to the various results are briefly summarized below:

- Studies described in [1]:
  - Experimental animals: male Wistar rats, 21 days-old with a mean body weight of 52.5 ± 3.1g.
  - Durations of treatments: 57 days.
  - Three experimental groups of rats with six animals per group:
    * The control group received an AIN-93G diet [29], where concentrations of vitamin A (4000 IU/kg) was replaced by an equivalent amount of β-carotene (14,400 μg/kg).
    * Vitamin A-deficient group received the AIN-93G without any source of vitamin A.
    * Vitamin A and iron-deficient group received the AIN-93G without any source of vitamin A and only 12.2 mg of iron/kg of diet.
- Studies described in [2]:
  - Experimental animals: male Wistar rats, 21 days-old with a mean body weight of 65.7 ± 5.5 g.
  - Duration of treatments: 59 days.
  - 5 experimental groups of rats with six animals per group:
    * The control group received the AIN-93G diet, containing 4000 IU of vitamin A/kg.
    * Vitamin A-deficient group received the AIN-93G diet without any source of vitamin A.
    * Iron-deficient group received the AIN-93G diet without any source of iron.
    * Vitamin A and iron-deficient group received the AIN-93G diet without any source of vitamin A or iron.
    * All-trans retinoic acid (atRA) group received the AIN-93G diet with 12 mg atRA/kg of diet.

Given these discrepancies (between the results presented in [1,2]), two different subprocesses were considered in the next stage of the analysis. The first one, in which vitamin A deficiency has an influence on the increase of BMP6, leading to hepcidin expression via HJV-BMP6-SMAD4 pathway (see subprocess (p)) in Figure 4). The second one, in which BMP6 induces SMAD7, which negatively regulates hepcidin (see subprocess (k)) in Figure 4). These two opposite mechanisms are included in the same number of t-clusters. Therefore, it seems they may be equally important for the modeled system, which does not solve the problem of existing different literature reports.

The results of the t-cluster analysis may be too general, thus performing clustering as the only analysis may lead to an omission of some meaningful relationships. Nevertheless, clustering may narrow the area of search and suggest a further direction of research. Therefore, the analysis of the average number of transition firings was performed for the mentioned before subprocesses (k) and (p).

The results of the analysis of the average number of firings of selected transitions are presented below:

- Subprocess stimulating hepcidin expression ($t_{83}$—VAD leads to increase of BMP6): the average number of firings is equal to 24.30.
- Subprocess inhibiting hepcidin expression ($t_{74}$—BMP6 induces SMAD7): the average number of firings is equal to 13.99.

This analysis showed that the elementary process associated with activating HJV-BMP6-SMAD4 pathway (which stimulates hepcidin expression) is more often fired than the subprocess leading to a negative regulation of hepcidin expression by involvement of BMP6 in SMAD7 induction.

## 4. Methods

Petri nets are mathematical objects that in a natural way can be used for modeling and analysis of systems composed of concurrent processes. Their origins are in the area of theoretical computer science, and for decades they were used mainly for modeling technical systems. However, during the last two decades, it appeared that they are very well suited for modeling and analysis of biological systems [10,11,12,13].

One of the reasons of this suitability is a structure of Petri nets, being a directed weighted bipartite graph. It means that a Petri net is composed of two disjoint sets of vertices. Vertices from one of these sets are called transitions and they usually correspond to some elementary active components of a modeled system (e.g., chemical reactions), while vertices from the other set, called places, are counterparts of elementary passive components of the system (e.g., substrates or products of chemical reactions). The vertices can be joined by an arc but only vertices of various types can be connected in this way. The arcs correspond to some causal relations between passive and active components of the net.

However, a Petri net is not a graph—there is one more, very important kind of components, i.e., tokens. They reside in places and can flow from one place to another through transitions. This flow is a crucial feature of Petri nets since it corresponds to flow of information, substances, signals, etc. through the modeled system. It is governed by a simple principle, called transition firing rule. According to it a transition becomes active if in every of places directly preceding it (such places are called pre-places of this transition) the numbers of tokens are equal to at least the weight of an arc joining a given place with the transition. An active transition can be fired, what means that tokens flow from its preplaces to its postplaces (i.e., places directly succeeding the transition), wherein the numbers of flowing tokens are equal to the weights of respective arcs.

Petri nets, as mathematical objects, can be described and analyzed using mathematical methods but they also have an intuitive and useful graphical representation. In this representation, transitions are depicted as rectangles or bars, places as circles, arcs as arrows, tokens as dots, or positive integer numbers located in places and weights as numbers labeling arcs (when a weight is equal to one, it is usually omitted in this representation for simplicity). The graphical representation is very helpful in understanding a structure of the modeled system and especially useful at the stage of developing the model and simulating it [10,13,22,30].

However, despite that the graphical representation of Petri nets is very useful, it is not well suited for a formal analysis of them. Hence, another representation is also often used, i.e., an incidence matrix. Such a matrix *A* is composed of *n* rows corresponding to places and *m* columns corresponding to transitions. Entry $a_{ij}$ of this matrix is a number equal to a difference between the numbers of tokens present in place $p_{i}$ before and after firing transition $t_{j}$.

An analysis of a Petri net-based model of a biological system can be based on t-invariants. Such an invariant is vector *x* being a solution of the following equation:

A·x=0.

The length of vector *x* is equal to *m*, i.e., the number of transitions, and each entry in this vector correspond to a transition. With t-invariant *x* there is associated a set of transition called its support. The elements of the support are transitions which correspond to positive entries in *x*. More formally, a support of t-invariant *x* is set $s\left(x\right)=\left\{t_{j}:x_{j}>0,j=1,2,\ldots ,m\right\}$.

t-invariants correspond to subprocesses, which can be especially important for the functioning of the modeled system. If every transition $t_{j}\in s\left(x\right)$ is fired $x_{j}$ times, then a distribution of tokens over the set of places (such a distribution is called a marking of a Petri net) does not change. The state of the modeled system becomes unchanged. Hence, t-invariants correspond to subprocesses which do not change the state of the modeled biological system [22]. Moreover, the Petri net model should be covered by t-invariants, i.e., each transition should belong to a support of at least one t-invariant. In a biological context, every elementary biological process modeled by a transition contributes to a behavior of the biological system. Therefore, the analysis of t-invariants may lead to a better understanding of interactions between the subprocesses of the modeled biological system. However, there is an assumption that the analyzed biological system is in a steady state.

There is also another type of sets of transitions important in the analysis of Petri net-based models of biological systems, i.e., Maximal Common Transition sets (MCT sets). Elements of such a set are transitions, which exclusively belong to supports of exactly the same t-invariants. From this follows that MCT sets divide the set of all transitions into disjoint subsets (i.e., every transition belongs to exactly one MCT set). It should be noted that some of MCT sets may contain only one transition—they are called trivial MCT sets. In general, MCT sets (except the trivial ones) correspond to some functional modules of the modeled biological system [13,22,31].

Apart from that transitions can be grouped into MCT-sets, also t-invariants can be grouped into sets called t-clusters. They contain those t-invariants which are similar to each other according to some similarity measure. Such clusters can be determined using standard clustering algorithms. However, there are many algorithms of this type and there are also many similarity measures which can be used. So, despite that to determine a collection of t-clusters standard algorithms and measures can be used, the task is not a trivial one, since the algorithm as well as the similarity measure should be properly chosen. Moreover, the number of clusters should also be determined in a proper way [21,32].

In addition to the mentioned analyzes, more detailed analyzes can be also carried out, i.e., a significance analysis with the completion by a knockout analysis and an analysis of an average number of transition firings [33]. The significance analysis allows determining a significance of a given elementary process based on the occurrence frequency of a transition (corresponding to such an elementary process) in all supports of t-invariants. The significance of a given transition is a ratio of the number of t-invariant supports containing this transition to the number of all t-invariants and it is expressed in percents.

The significance analysis is directly associated with the knockout analysis, which evaluates the significance of a given transition (or a set of transitions) based on the quantity of excluded subprocesses as a result of a knockout. A current number of t-invariants can be determined once again after the knockout of a selected transition. On this basis, it may be calculated how many subprocesses were excluded in consequence of the knockout. The more subprocesses were excluded, the more important the given elementary process is.

## 5. Conclusions

Using a systems approach based on Petri nets allows the analysis of biological phenomena as systems characterized by a complex net of interactions. To study the presented system in detail, the t-cluster analysis, the significance analysis, and the analysis of the average number of transition firings were performed. The above analyzes allowed to distinguish the most important mechanisms positively stimulating the expression of hepcidin and the most important mechanisms negatively regulating the expression of hepcidin. In addition, apart from distinguishing the most significant subprocesses/elementary processes for the functioning of the model, the average number of firings of particular transitions were also determined (it means that the frequency of occurrence of individual elementary processes).

**Figure 5:**
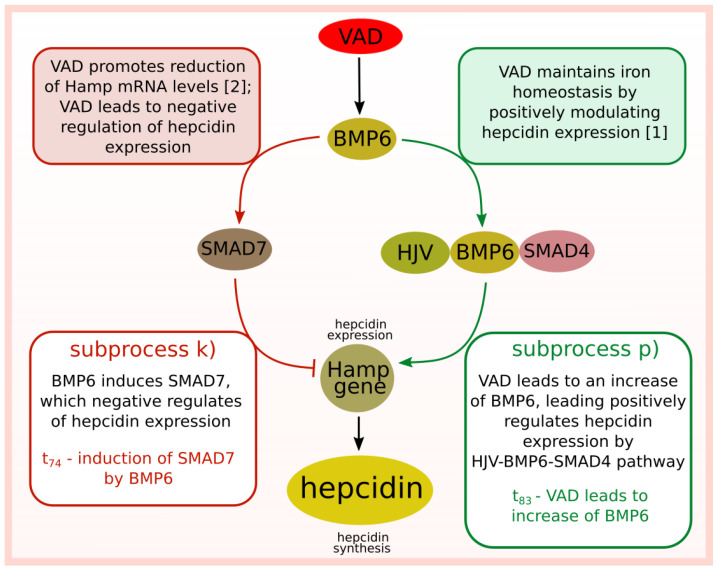
Diagram of two opposing mechanisms of regulation of hepcidin expression modulated by vitamin A deficiency.

In the case of the studied system focusing on the effect of vitamin A deficiency on the regulation of iron homeostasis, a thorough understanding of the biological problem is difficult due to contradictory literature information. The study of the proposed model aims to understand the analyzed system’s nature better and systematize the knowledge. The final analysis also allows resolving some doubts as to the discrepancy in literature reports, where VAD leads to negative regulation of hepcidin expression [2] (in this paper, the authors emphasize the fact that the achieved results are different than expected) or to positive regulation of hepcidin expression [1]. A graphical representation of this problem is shown in Figure 5, where the mechanism in which VAD negatively regulates hecidin expression is associated with induction of SMAD7 by BMP6, while the mechanism in which VAD positively regulates hecidin expression is associated with stimulation of HJV-BMP6-SMAD4 pathway. The t-cluster analysis showed that these two opposite subprocesses may be equally important for the functioning of the modeled system. However, the more detailed analyses showed differences. Both the significance analysis as well as the analysis of the average number of transition firings indicated that VAD more frequently positively stimulates hepcidin expression, and this mechanism is more significant than mechanism inhibiting hepcidin expression indirectly by VAD.

## Figures and Tables

**Table 1:** List of transitions.

ID	Biological Meaning	ID	Biological Meaning
$t_{0}$	cells involved in iron homeostasis	$t_{45}$	increase of DMT1 by apo-IRP1
$t_{1}$	hepcidin synthesis	$t_{46}$	inhibition of Ft by apo-IRP1
$t_{2}$	hepcidin binds to Fpn	$t_{47}$	inhibition of Fpn by apo-IRP1
$t_{3}$	hepcidin and Fpn binds JAK2	$t_{48}$	holo-IRP1 formation
$t_{4}$	source of JAK2	$t_{49}$	apo-IRP2 formation leads to the same effect as apo-IRP1
$t_{5}$	phosphorylation of Tyr residues in Fpn by JAK2	$t_{50}$	decrease of TfR1 caused by holo-IRP1
$t_{6}$	signal for displacement to cytoplasm	$t_{51}$	decrease of DMT1 caused by holo-IRP1
$t_{7}$	dephosphorylation of Fpn	$t_{52}$	increase of Ft caused by holo-IRP1
$t_{8}$	ubiquitination of Fpn	$t_{53}$	increase of Fpn caused by holo-IRP1
$t_{9}$	lysosomal degradation of Fpn	$t_{54}$	iron status
$t_{10}$	degradated Fpn prevents iron release from enterocytes and splenic macrophages	$t_{55}$	expression of hepcidin
$t_{11}$	iron accumulation lead to decrease of iron concentration in serum	$t_{56}$	inflammation
$t_{12}$	down regulation of DMT1 by blocked iron in enterocytes	$t_{57}$	IL-6 binds with JAK
$t_{13}$	source of ubiquitin protein E3	$t_{58}$	activation of STAT3
$t_{14}$	iron export to enterocytes by DMT1	$t_{59}$	expression of hepcidin by JAK-STAT3
$t_{15}$	source of DMT1	$t_{60}$	modulation of hepcidin expression
$t_{16}$	iron ion Fe${}^{3+}$ reduction to Fe${}^{2+}$ via Dcytb	$t_{61}$	Tf binds to TfR1 in case of normal iron status
$t_{17}$	source of Dcytb	$t_{62}$	HFE binds to TfR1 in case of iron deficiency
$t_{18}$	source of iron ion Fe${}^{3+}$	$t_{63}$	HFE binds to TfR1 and prevents Tf binding to TfR1 which decrease of hepcidin expression
$t_{19}$	down regulation of DMT1 leads to decrease of iron absorption	$t_{64}$	Tf binds to TfR1 and prevents HFE binding
$t_{20}$	Fenton reaction	$t_{65}$	mHJV acts as BMP cofactor
$t_{21}$	increase of oxidative stress via ROS	$t_{66}$	BMP6 binds to BMPR I and II
$t_{22}$	iron export from LIP to cells	$t_{67}$	source of BMPR I
$t_{23}$	absorption of iron	$t_{68}$	source of BMPR II
$t_{24}$	feedback iron form storage in enterocytes	$t_{69}$	BMP and BMPRs binding induces phosphoryla tion of BMPRs
$t_{25}$	iron export from enterocytes to blood circulation by Fpn	$t_{70}$	phosphorylation of SMAD 1,5 and 8
$t_{26}$	enterocytes ferroportin	$t_{71}$	phosphorylated SMAD 1,5 and 8 binds SMAD4
$t_{27}$	Fe${}^{2+}$ oxydation to Fe${}^{3+}$ by Heph	$t_{72}$	source of SMAD4
$t_{28}$	source of hephaestin	$t_{73}$	translocation to the nucleus
$t_{29}$	iron from enterocytes binds to Tf	$t_{74}$	BMP6 induces SMAD7
$t_{30}$	source of transferrin	$t_{75}$	negative ragulation of hepcidin
$t_{31}$	source of transferrin receptor protein 1	$t_{76}$	mHJV is located in membrane
$t_{32}$	iron absorption by mammals via endocytosis	$t_{77}$	activation of furin
$t_{33}$	IRP/IRE mechanism in iron overload	$t_{78}$	HJV is released from membrane via proteolytic reaction of furin
$t_{34}$	IRP/IRE mechanism in iron deficiency	$t_{79}$	sHJV blocks BMPRs
$t_{35}$	degradation of FBXL5	$t_{80}$	inhibition of hepcidin expression
$t_{36}$	increase of IRP2	$t_{81}$	vitamin A deficiency
$t_{37}$	increase of FBXL5	$t_{82}$	VAD leads to impairment of inflammatory respon se which leads to increase of cytokines
$t_{38}$	degradation of IRP2	$t_{83}$	VAD leads to increase of BMP6
$t_{39}$	apo-IRP1 formation	$t_{84}$	decrease of BMP6
$t_{40}$	apo-IRP1 binds to IRE in 3′UTR region of TfR1	$t_{85}$	decreased BMP6 inhibits BMP pathway and exp ression of hepcidin
$t_{41}$	apo-IRP1 binds to IRE in 3′UTR region of DMT1	$t_{86}$	impair of erythropoiesis
$t_{42}$	apo-IRP1 binds to IRE in 5′UTR region of Ft	$t_{87}$	increased phagocytosis of malformed and undiff erential erythrocytes
$t_{43}$	apo-IRP1 binds to IRE in 5′UTR region of Fpn	$t_{88}$	accumulation of iron in spleen
$t_{44}$	increase of TfR1 by apo-IRP1	$t_{89}$	decrease of HAMP mRNA level despite increase of BMP6

**Table 2:** List of nontrivial Maximal Common Transition (MCT) sets.

MCT Set	Contained Transitions	Biological Interpretation
$m_{1}$	$t_{0}$, $t_{1}$, $t_{2}$, $t_{3}$, $t_{4}$, $t_{5}$, $t_{6}$, $t_{7}$, $t_{8}$, $t_{9}$, $t_{10}$, $t_{13}$, $t_{14}$, $t_{16}$, $t_{17}$, $t_{54}$	Iron ion Fe${}^{3+}$ is reduced to ion Fe II by Dcytb and then is exported to enterocytes (to LIP) by DMT1. Hepcidin binds to Fpn and leads to degradation and internalization of Fpn. What results in prevention of iron release from enterocytes and macrophages.
$m_{2}$	$t_{66}$, $t_{67}$, $t_{68}$, $t_{69}$, $t_{70}$, $t_{71}$, $t_{72}$, $t_{73}$	Expression of hepcidin by HJV-BMP6-SMAD4 pathway.
$m_{3}$	$t_{23}$, $t_{25}$, $t_{27}$, $t_{28}$	Iron in enterocytes is absorbed by Ft and is also exported by Fpn to blood circulation.
$m_{4}$	$t_{57}$, $t_{58}$, $t_{59}$, $t_{60}$	Expression of hepcidin by cytokines IL-6 (JAK-STAT3 pathway) and IL-1β.
$m_{5}$	$t_{77}$, $t_{78}$, $t_{79}$, $t_{80}$	Low iron level activates furin which release HJV from membrane via proteolytic reaction. Soluble HJV blocks BMPRs and inhibits expression of hepcidin by inhibition of HJV-BMP6-SMAD4 pathway.
$m_{6}$	$t_{86}$, $t_{87}$, $t_{88}$, $t_{89}$	Vitamin A deficiency and iron deficiency impair of erythropoiesis, which results in increased phagocytosis of malformed and undifferential erythrocytes. This mechanism in consequence leads to accumulation of iron in spleen and to decrease of Hamp mRNA level.
$m_{7}$	$t_{35}$, $t_{36}$, $t_{37}$	Low iron level leads to degradation of FBXL5, which results in increase of IRP2. IRP2 play the same role as IRP1, leads to increase of TfR1 and DMT1 and to decrease of Ft and Fpn.
$m_{8}$	$t_{12}$, $t_{19}$	Hepcidin leads to degradation and internalization Fpn, which results in down-regulation of DMT1 and leads to decrease of iron absorption.
$m_{9}$	$t_{20}$, $t_{21}$	Iron is engaged in Fenton reaction, which leads to increase of oxidative stress via ROS.
$m_{10}$	$t_{29}$, $t_{32}$	Iron absorption by mammals via endocytosis. To be precise, iron ion Fe${}^{3+}$ binds to transferrin (Tf) and next binds to transferrin receptor protein 1 (TfR1), which results in iron absorption.
$m_{11}$	$t_{33}$, $t_{48}$	Formation of holo-IRP1 (IRP/IRE mechanisms) in case of increase of iron concentration.
$m_{12}$	$t_{34}$, $t_{39}$	Formation of apo-IRP1 (IRP/IRE mechanisms) in case of decrease of iron concentration.
$m_{13}$	$t_{40}$, $t_{44}$	apo-IRP1 binds to IRE in 3′UTR region of TfR1, which leads to increase of TfR1.
$m_{14}$	$t_{41}$, $t_{45}$	apo-IRP1 binds to IRE in 3′UTR region of DMT1, which leads to increase of DMT1.
$m_{15}$	$t_{42}$, $t_{46}$	apo-IRP1 binds to IRE in 5′UTR region of Ft, which leads to decrease of Ft.
$m_{16}$	$t_{43}$, $t_{47}$	apo-IRP1 binds to IRE in 5′UTR region of Fpn, which leads to decrease of Fpn.
$m_{17}$	$t_{61}$, $t_{64}$	In case of iron overload or iron normal status Tf bind to TfR1, which prevents HFE binding. Free HFE leads to increase of hepcidin expression.
$m_{18}$	$t_{62}$, $t_{63}$	In case of iron deficiency HFE binds to TfR1, which lead to decrease of hepcidin expression (and in consequence to increase of iron concentration).
$m_{19}$	$t_{74}$, $t_{75}$	BMP6 induces SMAD7, which negative regulates of hepcidin.
$m_{20}$	$t_{84}$, $t_{85}$	Low iron level and vitamin A deficiency lead to decrease of BMP6, which results in inhibition of HJV-BMP6-SMAD4 pathway.

**Table 3:** List of subprocesses included in t-clusters.

ID	Biological Meaning
(a)	Iron is exported by Fpn to blood circulation and then it can be absorbed by mammalian cells via endocytosis. To be precise, iron ion Fe${}^{3+}$ binds to transferrin (Tf) and next binds to transferrin receptor protein 1 (TfR1), which results in iron absorption.
(b)	Iron can be engaged in Fenton reaction, but it occurs rarer than iron absorption in enterocytes.
(c)	IRP/IRE mechanisms play important roles: in case of increase of iron concentration holo-IRP1 is formed and it leads to increase of Fpn and to decrease of DMT1 and TfR1.
(d)	Iron is exported to enterocytes by DMT1, where is absorbed by Ft.
(e)	IRP/IRE mechanisms play important roles: in case of decrease of iron concentration apo-IRP1 is formed and it leads to increase of DMT1 and TfR1 and to decrease of Fpn.
(f)	Expression of hepcidin by free HFE (in case of iron overload or iron normal status Tf bind to TfR1, which prevents HFE binding).
(g)	In case of iron deficiency HFE binds to TfR1, which lead to decrease of hepcidin expression.
(h)	Expression of hepcidin by cytokines IL-6 (JAK-STAT3 pathway) and IL-1β
(i)	Vitamin A deficiency additionally stimulates gene Hamp expression by increase of IL-6 and IL-1β.
(j)	Hepcidin binds to Fpn and leads to degradation and internalization of Fpn. This mechanism results in iron accumulation in enterocytes and macrophages and in consequence it leads to decrease of iron concentration in serum.
(k)	BMP6 induces SMAD7, which negative regulates of hepcidin expression ( HJV-BMP6-SMAD4 pathway inactive).
(l)	Inflammation leads to hepcidin expression by cytokines.
(m)	TfR1 is necessary to iron absorption by mammalian cells.
(n)	Expression of hepcidin by HJV-BMP6-SMAD4 pathway (HJV-BMP6-SMAD4 pathway leads to expression of Hamp gene).
(o)	Low iron level activates furin which release HJV from membrane via proteolytic reaction. Soluble HJV blocks BMPRs and inhibits expression of hepcidin by inhibition of HJV-BMP6-SMAD4 pathway.
(p)	Deficiency of vitamin A leads to increase of BMP6.
(r)	BAMP6 is engaged in HJV-BMP6-SMAD4 pathway, which leads to expression of Hamp gene.
(s)	Low iron level and vitamin A deficiency lead to decrease of BMP6, which results in inhibition of HJV-BMP6-SMAD4 pathway.
(t)	Vitamin A deficiency and iron deficiency impair of erythropoiesis, which results in increased phagocytosis of malformed and undifferential erythrocytes. This mechanism in consequence leads to accumulation of iron in spleen and to decrease of Hamp mRNA level.

**Table 4:** Results of significance analysis for selected elementary processes associated with stimulation and inhibition of hepcidin expression.

Subprocess	ID	Name of Elementary Process	Significance		AvgT
			Frequency Trans./t-inv.	Percentage Ratio [%]	
**Subprocesses Associated with Stimulation of Hepcidin Expression**					
HJV-BMP6-SMAD4 pathway	$t_{73}$	translocation to the nucleus	118/402	29%	13.98
Cytokines IL-6 and IL-1β	$t_{59}$	expression of hepcidin by JAK STAT3	252/402	63%	46.94
	$t_{60}$	modulation of hepcidin expression	252/402	63%	49.86
	$t_{82}$	VAD leads to impairment of inflammatory response which leads to increase of cytokines	126/402	31%	24.38
Free HFE	$t_{55}$	expression of hepcidin	40/402	10%	5.80
	$t_{64}$	Tf binds to TfR1 and prevents HFE binding	106/402	26%	6.01
**Subprocesses associated with inhibition of hepcidin expression**					
Inhibition of HJV-BMP6-SMAD4 pathway	$t_{80}$	inhibition of hepcidin expression	48/402	12%	2.00
	$t_{85}$	decreased BMP6 inhibits BMP pathway and expression of hepcidin	78/402	19%	0.26
Binding HFE to TfR1	$t_{62}$	HFE binds to TfR1 in case of iron deficiency	68/402	17%	0.19
	$t_{63}$	HFE binds to TfR1 and prevents Tf binding to TfR1 which decrease of hepcidin expression	68/402	17%	0.19
Induction SMAD7 by BMP6	$t_{74}$	BMP6 induces SMAD7	41/402	10%	13.99
	$t_{75}$	negative ragulation of hepcidin	41/402	10%	13.96
Impairing of erythropoiesis	$t_{86}$	impair of erythropoiesis	38/402	9%	0.89

## Data Availability

Not applicable.

## Supplementary Materials

The following are available online at https://www.mdpi.com/article/10.3390/ijms23031189/s1.

## Abbreviations

The following abbreviations are used in this manuscript:

atRA	all-trans retinoic acid
BMP	bone morphogenetic protein
BMP6	bone morphogenetic protein 6
BMPRs	bone morphogenetic protein receptors
C–H	Calinski–Harabasz coefficient
Dcytb	duodenal cytochrome b
DMT1	divalent metal transporter 1
EPO	erytropoietin
FBXL5	F-box and leucine rich repeat protein 5
FeD	iron deficiency
FPN	ferroportin
Ft	ferritin
HAMP	hepcidin antimicrobial peptide (gene encoding hepcidin)
Heph	hephaestin
HFE	homeostatic iron regulator
HJV	hemojuvelin
IL-6	interleukin-6
IL-1β	interleukin-1β
IRE	iron responsive element
IRP	iron regulatory protein
JAK2	janus kinases
LIP	labile iron pool
MCT sets	Maximal Common Transition sets
mHJV	membrane hemojuvelin
MSS	Mean Split Silhouette index
ROS	reactive oxygen species
sHJV	soluble hemojuvelin
SMAD	mothers against decapentaplegic
STAT	signal transducer and activator of transcription protein
Tf	transferrin
TfR1	transferrin receptor protein 1
trans./t-inv.	transition/t-invariant
Tyr	tyrosine
VAD	vitamin A deficiency
